# Capillary Electrophoresis Hyphenated with Mass Spectrometry for Determination of Inflammatory Bowel Disease Drugs in Clinical Urine Samples

**DOI:** 10.3390/molecules22111973

**Published:** 2017-11-15

**Authors:** Katarína Maráková, Juraj Piešťanský, Zuzana Zelinková, Peter Mikuš

**Affiliations:** 1 Department of Pharmaceutical Analysis and Nuclear Pharmacy, Faculty of Pharmacy, Comenius University in Bratislava, Odbojárov 10, SK-832 32 Bratislava, Slovakia; marakova@fpharm.uniba.sk (K.M.); piestansky@fpharm.uniba.sk (J.P.); 2 Toxicological and Antidoping Center (TAC), Faculty of Pharmacy, Comenius University in Bratislava, Odbojárov 10, SK-832 32 Bratislava, Slovakia; 3 Department of Gastroenterology, St Michael’s Hospital, Satinského 1, SK-811 08 Bratislava, Slovakia; zdetkova@yahoo.co.uk

**Keywords:** thiopurines, human urine, capillary electrophoresis, drug analysis, mass spectrometry, inflammatory bowel disease

## Abstract

Azathioprine is the main thiopurine drug used in the treatment of immune-based inflammations of gastrointestinal tract. For the purpose of therapy control and optimization, effective and reliable analytical methods for a rapid drug monitoring in biological fluids are essential. Here, we developed a separation method based on the capillary electrophoresis (CE) hyphenated with tandem mass spectrometry (MS/MS) for the simultaneous determination of azathioprine and its selected metabolites (6-thioguanine, 6-mercaptopurine, and 6-methylmercaptopurine) as well as other co-medicated drugs (mesalazine, prednisone, and allopurinol). The optimized CE-MS/MS conditions provided a very efficient and stable system for the separation and sensitive detection of these drugs in human urine matrices. The developed method was successfully applied for the assay of the targeted drugs and their selected metabolites in urine samples collected from patients suffering from inflammatory bowel disease (IBD) and receiving azathioprine therapy. The developed CE-MS/MS method, due to its reliability, short analysis time, production of complex clinical profiles, and favorable performance parameters, evaluated according to FDA guidelines for bioanalytical method validation, is proposed for routine clinical laboratories to optimize thiopurine therapy, estimate enzymatic activity, and control patient compliance with medication and co-medication.

## 1. Introduction

Crohn’s disease (CD) along with ulcerative colitis belong to the group of inflammatory bowel diseases (IBDs) characterized by chronic inflammation of gastrointestinal tract (GIT) based on immune-mediated reaction. IBDs affect mainly young patients (between the ages 15–30) and their incidence is still rising. Even though there is currently no cure for Crohn’s disease, a wide range of treatment options is available that can help to control and reduce the symptoms and complications and achieve and maintain a remission [1].

Thiopurines, namely azathioprine (AZA) and its main active metabolites (6-mercaptopurine, 6-MP, and 6-thioguanine, 6-TG), represent the main group of therapeutics used for Crohn’s disease treatment. Their pharmacological effect is based on the cytotoxic and immune suppressive function through various active metabolites that are created during complex enzymatic reactions [2]. For enhancing therapeutic effect, thiopurines are very often used in co-medication with other therapeutics, such as corticosteroids (prednisone, PRE, strong anti-inflammatory agent), nonsteroidal anti-inflammatory drugs (mesalazine, 5-amino-salicylic acid, MSL, potent scavenger of reactive oxygen species), and enzyme inhibitors (allopurinol, ALP, inhibitor of xanthine oxidase). 

Clinical use of thiopurine drugs is associated with various adverse effects (including hepatotoxicity) and with the resistance for thiopurine therapy in some patients. Inter-individual variability in therapeutic response could be attributed to several factors including genetic polymorphisms, differences in the enzymatic metabolism of thiopurines, comorbidities, compliance issues, use of concomitant medication, and drug pharmacokinetics (absorption, distribution, metabolism, and elimination) [3]. Due to the increasing number of IBD patients and the costs of biological therapy, monitoring thiopurine treatment is of a current importance. It can prevent or decrease the thiopurine therapy failure and help provide safe and effective therapeutic doses for a given patient. Moreover, it has a cost-saving potential. For this purpose, highly reliable and effective analytical methods are required enabling the separation of targeted drugs and their metabolites in complex biological matrices.

Thiopurines are determined in biological fluids most frequently by liquid chromatography (LC). LC–mass spectrometry (MS) (LC-MS) was applied for the determination of 6-TG and 6-methylmercaptopurine (6-MMP) in whole blood samples [4], and 6-MP and its metabolite 6-TG in human plasma [5,6]. ALP and its active metabolite oxypurinol (OXP) have been simultaneously determined via LC-MS/MS in human plasma [7,8,9] and in human urine [9,10]. MSL and its metabolite *N*-acetylmesalazine have been analyzed via LC-MS/MS in human plasma [11,12]. 

Separation techniques based on the capillary electrophoresis (CE) can provide several important advantages in comparison with well-established and universal LC techniques, such as high separation efficiency, rapid analysis, low costs, flexibility, low sample and electrolyte consumption, and minimum sample preparation. A unique electrophoretic separation mechanism provides a remarkable resolution power toward ionizable and ionic analytes [13,14,15]. Moreover, CE separation of charged compounds is compatible with advanced detection techniques requiring charged analytes, such as MS.

CE combined with MS, in comparison with the conventional detection techniques (UV, conductivity), enhances sensitivity and selectivity, which are crucial when analyzing drugs and metabolites in biological matrices. This is even more pronounced when applying a multiple reaction monitoring mode (MRM) in a tandem mass spectrometry (MS/MS). It allows a highly specific monitoring of particular ion transitions and fragments for each analyzed compound with a favorable signal-to-noise ratio. MS/MS also provides the structural characterization of analyzed compounds via their characteristic fragmentation mass spectra [15,16,17,18].

Several CE methods for the analysis of the drugs used in Crohn’s disease treatment have already been developed in various biological matrices. CE-UV methods have been applied for 6-MP and its metabolites (6-MMP and 6-TG) in plasma [19], 6-TG in urine [20], and ALP and its active metabolite OXP in serum [21] and urine [22]. CE with amperometric detection was applied for ALP and OXP in urine [23]. CE-MS was recently used for the group of IBD drugs present in commercial pharmaceuticals [24]. To our best knowledge, however, no CE-MS method has been developed for biological matrices containing the drugs used in Crohn’s disease treatment so far.

Therefore, this work aims to examine the possibilities of tandem mass spectrometry (MS/MS) as a CE detection step for the simultaneous analysis of the main chemical drugs currently used in IBDs (three thiopurines, PRE, MSL, ALP) in a real biological matrix (human urine) for current clinical applications.

## 2. Results and Discussion

### 2.1. Development of the CE-ESI-MS/MS Method

The CE-MS analytical conditions optimized in our previous work for pharmaceutical matrices [24] were properly modified and adapted here in order to achieve simultaneous separation and sensitive detection of the drugs used in Crohn’s disease therapy and their major metabolites in human urine. These are, namely, azathioprine, 6-thioguanine, 6-mercaptopurine, 6-methylmercaptopurine, mesalazine, prednisone, and allopurinol. 

In the first step, CE separation conditions were examined. Injecting spiked urine samples as models, a worse reproducibility of the analyses was observed, probably due to a modification of the inner capillary surface and, by that, electroosmotic flow (EOF) by constituents of the variable multicomponent matrix. This was why the capillary with 0.1 M NaOH was washed for 2 min before each analysis. This preconditioning step improved the reproducibility of CE separations (migration times) as well as the response of the MS detector (intensity of analytical signals). A 10 mM ammonium acetate adjusted at pH 9 by 3% (*v/v*) ammonium hydroxide and including a 5% (*v/v*) methanol addition was used as an optimum background electrolyte (BGE). It provided a baseline separation of all analytes in one run with maximum intensity of the detection signal. 

In the second step, ESI-MS/MS conditions were examined with spiked urine samples. The optimized parameters, summarized in Section 3, allowed the simultaneous analysis of all studied drugs present in the urine matrices without any significant separation and detection interferences. The MRM mode provided a favorable quantitation of the analytes with an acceptable signal-to-noise ratio even in the urine matrices. The optimum parent–product ion transitions of the studied drugs and their selected metabolites analyzed as the standards are listed in Table 1.

The effect of dilution of urine samples (i.e., matrix effect) on the MS detection signal was tested in the interval of 2–20-fold urine dilution. A more than 15% decrease in the analytical signal was found when analyzing the same concentration of analytes in the concentrated urine (max. 2–5-fold diluted) in comparison with the more diluted one (10–20-fold diluted). A 10-fold diluted urine compromised an acceptable ionization efficiency and detection response (due to low matrix interferences in the ESI source), with a good reproducibility of the analyses obtained with higher dilution on one side and lower limits of detection obtainable with lower dilution on the other side. Therefore, it was selected as an optimum dilution of the human urine samples for biomedical applications.

**Figure 1:**
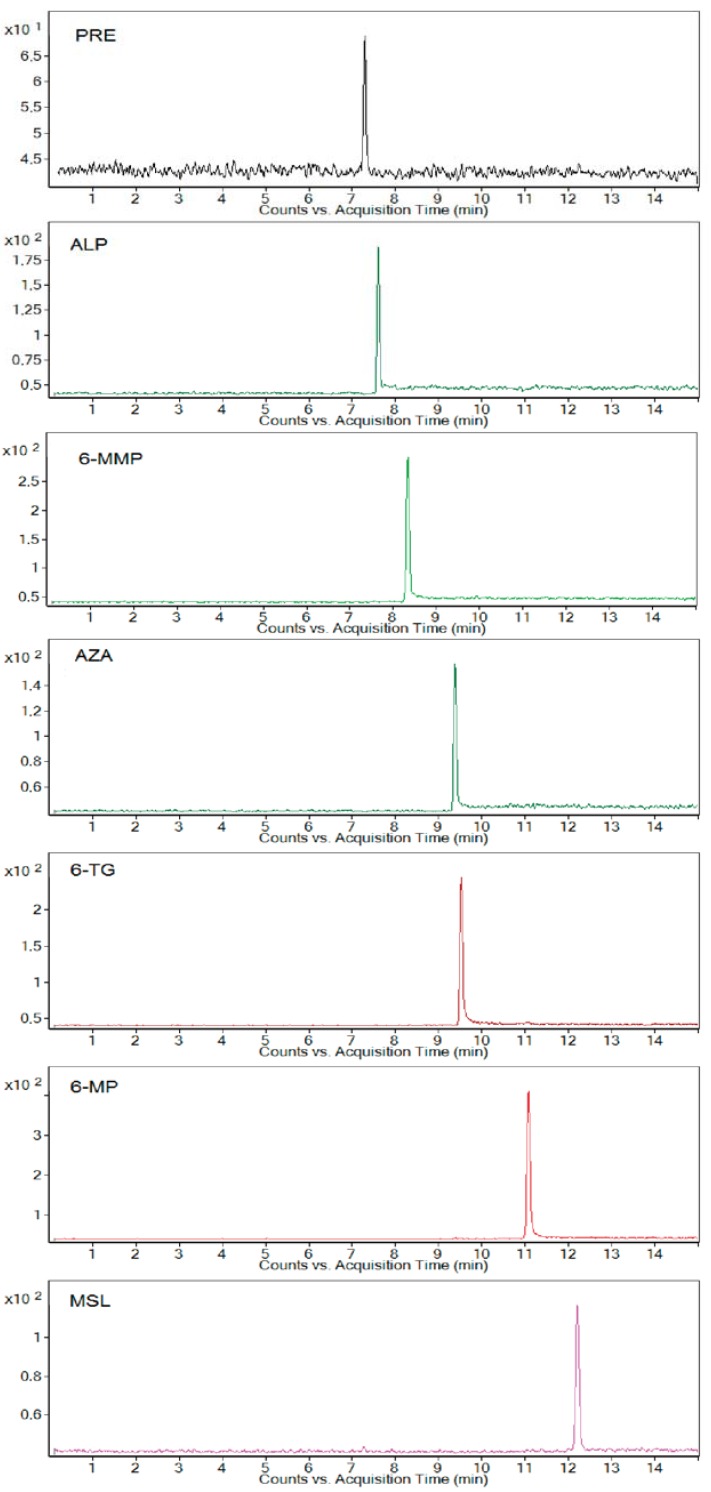
Analysis of model human urine sample. Multiple reaction monitoring mode (MRM) transitions from CE-ESI-MS/MS analysis of 10-fold diluted blank urine sample spiked with the mixed standard solution (concentration level of all analytes was 2.5 μg·mL^−1^). For the preparation of the sample and other working conditions, see Section 3.

Extracted electrophoreograms illustrating the simultaneous analysis of all studied standard drugs and metabolites spiked in a 10-fold-diluted blank urine sample under optimum separating conditions are shown in Figure 1.

### 2.2. Data Evaluation and Performance Parameters

The performance parameters of the optimized CE-ESI-MS/MS method were evaluated according to the Food and Drug Administration (FDA) Guidance for Industry, Bioanalytical Method Validation [25], and the resulting data are given in Table 2 and Table 3. The standard solutions of the drugs (for their preparation, see Section 3) were used for the validation experiments. The parameters of calibration lines were calculated using Microsoft Excel 2010 (Microsoft Corporation, Redmond, WA, USA).

To test the selectivity, i.e., an ability of the method to differentiate and quantify studied analytes in the presence of other components in the sample, blank human urine samples from three healthy volunteers were tested for the presence of interfering peaks. No interfering endogenous species were observed at the migration times of the targeted analytes when analyzing them at lower limit of quantification (LLOQ) concentration levels. This indicated that the proposed method is selective enough for the simultaneous analysis of the studied drugs and their selected metabolites in human urine matrices.

For the calibration curve, six calibration points were measured in triplicates. Good linear responses (determination coefficients ranging in the interval of 0.9979–0.9996) were obtained for the studied analytes in the concentration range of 0.25–15 μg·mL^−1^ (prednisone in the range of 0.5–15 μg·mL^−1^). According to FDA, the precision (RSD, %) and accuracy (RE, %) values should be ≤15% for all standards at all calibration levels except for LLOQ level where the determined values cannot exceed 20%. The back-calculated calibration points showed RSD values ranging from 0.18 to 10.31% for all analyzed substances. The percentage difference (RE) between the standard concentrations calculated from the calibration curves and the theoretical ones ranged from 0.06 to 14.86% for all standards at all calibration levels, so the FDA criteria were met.

The LLOQ represented the lowest point of calibration curve. LLOQ is defined as the lowest analyte concentration corresponding to a response at least 5 times higher than the blank response and which can be determined with 80–120% accuracy and 20% precision. LLOQ for prednisone and other analytes in urine were 0.5 μg·mL^−1^ and 0.25 μg·mL^−1^, respectively. The mean signal-to-noise ratio (S/N) values for LLOQ were in the range of 5.3–26.4 for all studied drugs, and precision and accuracy being in the range of 4.20–14.97% and 4.13–18.74%, respectively. Limits of detection (LODs, S/N = 3) of the analytes in human urine ranged in the interval of 0.0284–0.268 μg·mL^−1^. Hence, the estimated LODs demonstrated high sensitivity of the proposed method, and LLOQs were suitable for the reliable quantification of the trace concentration levels of the studied drugs in human urine samples.

To evaluate the precision and accuracy of the proposed method, calculated as the relative standard deviations (RSD, %) and relative errors (RE, %), respectively, the spiked urine samples (QC samples) at three concentration levels (low, medium, and high) were prepared and analyzed on the same day and over three consecutive days. The corresponding data are listed in Table 3. The RE values ranged in the interval of 0.06–18.74%. The RSD values for the intra- and inter-day precision were within the ranges of 0.34–14.97% and 1.34–15.84%, respectively. The determined values, accomplishing the FDA criteria (15–20%), clearly confirmed acceptable precision and accuracy of the developed method.

It is well known that during the ESI process co-eluting matrix components can influence the recovery of analyte ion production by competition processes (ion suppression or enhancement). To evaluate the recovery and the effect of co-eluting matrix components, the peak areas of the analytes in the spiked urine samples were compared with those of corresponding standard solutions in water at equivalent concentrations. The recoveries of all analytes at three concentration levels (QC samples) were in the range of 83.04–114.2%, with the precisions ranging in the interval of 0.28–9.16%. The recovery values (Table 3) indicated an acceptable influence of the matrix on the detection response of the analytes. Hence, the proposed method is suitable for the analysis of the selected drugs and their metabolites in human urine matrices. 

The stability of stock solutions and working solutions was examined by measuring them after 3 days of storage at 4 °C and after 6 h storage at room temperature. The fluctuations of measured concentrations of all analytes were in the range of 95.1–102.5% to the initial concentrations. The stability of the analytes in urine matrices was tested after storing the QC samples for 24 h at room temperature (short-term stability) and after performing three complete freeze/thaw cycles from −18 to +25 °C (freeze/thaw stability). The obtained relative errors were less than 12.69% for all analytes. Hence, the stability results obtained were within the FDA acceptance criteria (15%), and the proposed urine sample handling and preparation is suitable for the routine use. 

### 2.3. Application of the CE-ESI-MS/MS Method

The optimized and validated CE-ESI-MS/MS method was applied in the clinical analysis to measure urinary concentrations of the excreted drugs and their selected metabolites. The assay was carried out in a group of 13 patients (four men and nine women) suffering from the Crohn’s disease and treated with the azathioprine drug (daily dosage of 50–125 mg). Some of the patients received mesalazine as a co-medication too. All subjects gave their informed consent for inclusion before they participated in the study. The study was conducted in accordance with the Declaration of Helsinki, and the protocol NsM 14-54/2016 was approved by the Medical Ethical Committee of St Michael’s Hospital, Bratislava, Slovakia.

**Figure 2:**
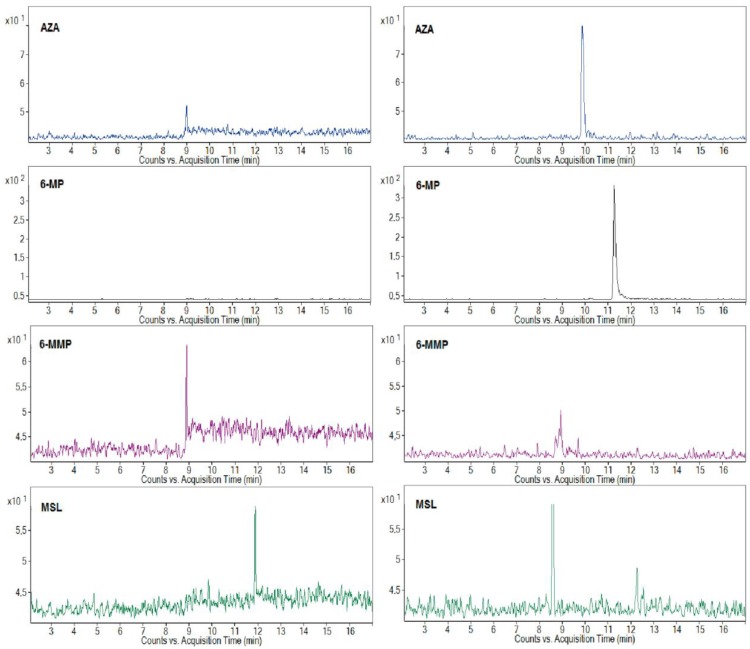
Analysis of clinical human urine sample. MRM transitions from CE-ESI-MS/MS analysis of 10-fold diluted human urine sample were obtained from the patients treated by azathioprine with a daily dosage of 50 mg (**left** panel) and 100 mg (**right** panel), and a mesalazine co-medication. For the preparation of the sample and other working conditions, see Section 3.

The representative MRM profiles from the CE-ESI-MS/MS analysis of the clinical urine samples are shown in Figure 2. The profiles in the left and right panels represent the results of two randomly selected patients with a 50 and 100 mg AZA dosage, respectively, and MSL co-medication. The MRM profiles with the selected quantifier signals, and the fact that there were no significant interfering peaks in the selected transitions, were favorable for the sensitive and reliable determination of the AZA and its metabolites, 6-MP, 6-MMP, and co-medicated drug MSL, in the clinical urine samples. According to the drug producer’s information, a 50% amount of the peroral dose of AZA is eliminated in urine in the first 24 h, and less than 2% is unchanged AZA and its major metabolite 6-MP. The typical content of AZA in the urine samples found by the proposed method was for the tested group of the patients in the concentration range of 1.29–14.85 μg·mL^−1^ (depending on the dose and metabolism of individual patients). According to the knowledge of many clinicians, therapy with some patients treated at home can be problematic due to their poor medication compliance. The developed CE-MS/MS method was successfully used to control patient compliance with thiopurine medication and relevant co-medication. The obtained peak responses to AZA in the MRM profiles, even if partially influenced by individual metabolisms, were consistent with the AZA dose administered to particular patients. Although it was less pronounced/consistent for the co-medicated MSL, probably because of higher variability in the creation of *N*-acetyl-mesalazine as its main metabolite (standard was not available, e.g., to confirm the peak eluting in 8.5 min in the right MSL panel in Figure 2), it was still sufficient for the reliable monitoring of MSL eliminated in urine after its peroral administration.

Thiopurine *S*-methyltransferase (TPMT) plays an important role in the metabolism of thiopurines used in IBD therapy, i.e., in keeping balance between active (non-methylated) and non-active (methylated) thiopurine metabolites. The proposed method allowed for the evaluation of three analytes (AZA, 6-MP, and 6-MMP) included in a detailed investigation of TPMT activity. It can be seen from the illustrative profiles in Figure 2 that a 6-MP/6-MMP ratio can be easily and reliably determined in different clinical urine samples. A great advantage of such monitoring is (i) its complexity (we can correlate an amount of the original drug AZA with its active, 6-MP, and inactive, 6-MMP, metabolites) and (ii) its non-invasive character (replacing blood for urine samples). This monitoring can be used continuously during thiopurine therapy without an excessive burdening of the patients. Based on the TPMT activity results, the patients can be properly treated by precisely increasing or decreasing their AZA dose or by a suitable co-medication that influences the activity of TPMT (e.g., ALP). To determine all practical benefits of such monitoring, large groups of properly treated patients must be clinically studied and statistical significance in the resulting data must be found.

## 3. Materials and Methods

### 3.1. Instrumentation

The capillary electrophoretic analyzer, Agilent 7100 Capillary Electrophoresis System (Agilent Technologies, Santa Clara, CA, USA), was used in this work to perform capillary zone electrophoresis (CZE). Data acquisition was controlled by Agilent ChemStation Softwave B.04.03 (Agilent Technologies, Santa Clara, CA, USA). Analyses were carried out in a 50 μm inner diameter (I.D.) uncoated fused silica capillary tube with an 85 cm total length in the cationic separation regime (progressively applied separation voltage of +30 kV) and at a constant temperature of 20 °C. Before first use, the capillary was conditioned by rinsing with 1 M NaOH for 5 min, with deionized water for 5 min, and finally with background electrolyte (BGE) for 20 min. Between the measurements, the preconditioning steps included an application of negative voltage (−25 kV) for 30 s and the flushing of the capillary with 0.1 M NaOH, water, and BGE (each for 120 s). The preconditioning was sufficient to restore and reequilibrate the capillary surface between the analyses and to achieve better reproducibility. The samples were injected hydrodynamically by applying a pressure of 50 mbar for 10 s, and, after the injection of the sample, the small zone of BGE (50 mbar for 2 s) was injected in order to ensure peak area reproducibility and a quantitative injection of the sample. A 10 mM ammonium acetate adjusted at pH 9 by 3% (*v/v*) ammonium hydroxide and including a 5% (*v/v*) methanol addition was used as an optimum background electrolyte (BGE). The resulting current was fixed at 10 μA. The proposed BGE was prepared daily and was renewed in the vials after every three runs. 

A tandem mass spectrometer, Agilent 6410 Series Triple Quadrupole (Agilent Technologies, Santa Clara, CA, USA), equipped with an electrospray ionization source (ESI), was used as a detection system in this work. The MS system operation and data acquisition were performed using Mass Hunter Work Station B.03.01 (Agilent Technologies, Santa Clara, CA, USA). CE-ESI-MS/MS coupling was carried out using a sheath liquid coaxial interface (Agilent Technologies, Santa Clara, CA, USA). A sheath liquid-based ESI interface is relatively robust and easy to implement in CE-MS/MS. The ionization of the studied drugs was carried out in positive ion mode. The optimum composition of the sheath liquid was 50% (*v/v*) methanol/water with an addition of 5 mM ammonium acetate (compromising a maximum intensity and reproducibility of the detection response for the drugs). The sheath liquid was delivered by a pump Agilent 1260 Infinity (Agilent Technologies, Santa Clara, CA, USA) and flowed through a splitter set at a ratio of 1:100 into the sprayer. The optimum sheath liquid flow rate was set at 8 μL·min^−1^ (the best compromise between a stable separation current and high sensitivity).

The sensitivity of the MS detection is highly dependent on several parameters related to the ESI-MS interface, such as nebulizing gas pressure, drying gas temperature and flow rate, capillary voltage, and protruding length of the CE capillary from the injection sprayer. The nebulizing gas (N_2_) flowed through the outer capillary channels under the pressure set to 10 psi. The drying gas temperature was 300 °C and its flow rate was 7 L·min^−1^. The capillary voltage of +4500 V was set in the MS detector. The optimal protruding length of the CE capillary from the interface was about 0.2–0.3 mm.

### 3.2. Chemicals and Samples

The CE electrolyte solution was prepared from ammonium acetate adjusted to pH 9.0 by 3% (*v/v*) ammonium hydroxide and with an addition of 5% (*v/v*) methanol; all chemicals were obtained from Sigma-Aldrich (Sigma-Aldrich, Steinheim, Germany). The used chemicals were of analytical or LC-MS grade. Demineralized water for the preparation of electrolyte and sample solutions was produced by a Millipore Simplicity 185 (UV) water purification system (Millipore, Molsheim, France). The electrolyte solution was filtered before use through nylon membrane filters of a 0.22 μm pore size (Millipore, Molsheim, France).

The drug standards, namely, prednisone, 6-thioguanine, 6-mercaptopurine monohydrate, allopurinol, azathioprine, mesalazine, and 6-methylmercaptopurine were purchased from Sigma-Aldrich (Sigma-Aldrich, Steinheim, Germany). 

### 3.3. Preparation of the Standard Solutions and Model Urine Samples

The stock standard solutions of the drugs were prepared in 10 mL amber volumetric flasks by dissolving 1 mg of the pure standard substance in demineralized water. All stock solutions were stored protected from the light in the refrigerator at 4 °C. It was found out in our previous work [24] that the presence of a 20% methanol in the samples improved the peak shape (avoiding a peak tailing) and, by that, improved the separation efficiency. For that reason, the working solutions of the drugs were prepared in a 20% (*v/v*) methanolic solution by proper dilution of the stock standard solutions or by spiking the stock standard solutions into biological samples (human urine).

The six concentration levels of the drugs in the injected model urine calibration solutions, prepared by spiking 10-fold diluted blank urine, were in the range of 0.5–15 μg·mL^−1^ and 0.25–15 μg·mL^−1^ for prednisone and the other studied drugs, respectively. Each calibration point was measured three times. The lowest concentration values of the drugs in the model urine calibration solutions corresponded to the LLOQs (the analyte response at the LLOQ level should be at least five times the response compared to blank one).

Quality control samples (QCs) at three concentration levels (0.5, 2.5, and 10 μg·mL^−1^ and 0.25, 2.5 and 10 μg·mL^−1^ for prednisone and the other drugs, respectively), were prepared in 10-fold diluted blank human urine. The prepared QCs correspond to low, medium, and high QC concentration levels. Each QC concentration level was measured five times.

### 3.4. Urine Samples Preparation

The model and real urine samples were obtained from three healthy volunteers and 13 patients suffering from Crohn’s disease and treated by azathioprine (the dosage was 50–125 mg of azathioprine per day), respectively. Each urine sample was divided into several vials and frozen (−18 °C) immediately after the sampling and stored in the freezer until use. The urine samples were thawed out at the laboratory temperature before use. All urine samples were centrifuged, filtered with 0.22 μm pore size nylon syringe filters (Millipore, Molsheim, France), appropriately diluted into the 20% (*v/v*) methanolic solution, and immediately injected into the electrophoretic analyzer.

## 4. Conclusions

This work demonstrated the suitability of the hyphenated CE-ESI-MS/MS method for the analysis of thiopurine drugs and their selected metabolites as well as other drugs currently used as co-medications for Crohn’s disease treatment in multicomponent biological matrices such as human urine. The proposed method represents the first CE-MS approach employed in clinical profiling of thiopurines so far.

The orthogonality of CE-ESI-MS/MS, based on the on-line combination of the electrophoretic separation mechanism (providing resolution of the analytes in time) and MS/MS (providing specific and highly sensitive detection of the targeted analyte fragments—Identifiers and quantifiers in the timely resolved mass profiles) was considerably enhanced over conventional CE-UV. Such conditions were reflected in favorable performance parameters evaluated according to the FDA guidelines for bioanalytical method validation. Along with this, the proposed CE-ESI-MS/MS method is a simple and fast analytical procedure (direct injection of diluted urine samples, relatively short analysis time), requires minimum sample and electrolyte consumption, and has lower performance and equipment costs (in comparison with HPLC-ESI-MS/MS). These parameters clearly indicated a suitability of the proposed method for routine control laboratories and for clinical use.

The application potential of the proposed method was illustrated by the reliable monitoring of metabolic profiles of AZA with or without co-medications (here, MSL) in a small group of patients suffering from Crohn’s disease. The ability to determine concentrations of AZA and its active as well as non-active metabolites (6-MP and 6-MMP) and other co-medicated drugs eliminated in urine could help elucidate the pharmacokinetics and in turn the factors that may contribute to the inter-individual variability in response to the drugs. This analytical approach could then be a useful tool to optimize and individualize thiopurine therapy (i.e., increase or decrease the AZA dose, or add a suitable co-medication affecting the activity of TPMT involved in thiopurine metabolism).

## Figures and Tables

**Table 1:** Parent–product ion transitions.

Drug	Parent Ion [M + H]^+^	Fragmentor Voltage (V)	Product Ion (Quantifier)	Product Ion (Qualifier)	Collision Energy (eV)
azathioprine	278.1	100	142.1	232.2	8
6-mercaptopurine	153.0	140	118.9	92.0	25
6-methylmercaptopurine	166.8	120	124.8	151.8	30
6-thioguanine	168.0	120	151.0	134.0	20
allopurinol	137.1	120	54.0	110.1	30
mesalazine	154.1	100	136.1	108.0	10
prednisone	359.2	120	147.1	237.1	20

**Table 2:** Performance parameters of the CE-ESI-MS/MS method ^a^.

Drug	t_m_ (min) ^b^ n = 15	RSD_tm_ (%) n = 15	Linear Range (μg·mL^−1^)	Intercept *a* (Counts min)	Slope *b* (Counts min μg ^−1^ mL)	**r^2^**	**LOD (μg·mL^−1^)**
azathioprine	9.377	0.57	0.25–15	13.72	163.8	0.9979	0.0547
6-mercaptopurine	11.058	0.84	0.25–15	25.40	660.4	0.9995	0.0284
6-methylmercaptopurine	8.317	0.77	0.25–15	44.13	389.6	0.9989	0.0560
6-thioguanine	9.494	1.01	0.25–15	−6.472	370.9	0.9996	0.0475
allopurinol	7.593	0.25	0.25–15	24.45	171.2	0.9996	0.134
mesalazine	12.239	0.17	0.25–15	17.51	152.5	0.9995	0.142
prednisone	7.379	1.47	0.5–15	13.33	38.87	0.9981	0.268

^a^ For the separation and other working conditions see Section 3. ^b^ Measured at three concentration levels, each sample five times.

**Table 3:** Evaluation of spiked urine samples (QC samples) for estimation of precision and accuracy of the CE-ESI-MS/MS method ^a^.

**Drug**	QC Low				QC Medium				QC High			
	**Accuracy (%)**	**Intra-Day Precision RSD (%)**	Inter-Day Precision ^b^ RSD (%)	Recovery (%)	Accuracy (%)	Intra-Day Precision RSD (%)	Inter-Day Precision ^b^ RSD (%)	Recovery (%)	Accuracy (%)	Intra-Day Precision RSD (%)	Inter-Day Precision ^b^ RSD (%)	Recovery (%)
AZA	118.8	5.18	6.64	110.7 ± 1.53	108.51	7.00	5.14	89.92 ± 4.08	100.9	1.45	6.81	96.14 ± 1.21
6-MP	95.87	4.21	8.91	107.6 ± 0.28	100.7	2.80	3.99	86.69 ± 1.91	100.4	1.04	6.56	98.02 ± 4.45
6-MMP	84.41	8.28	10.50	110.4 ± 9.16	106.4	0.81	9.38	87.99 ± 1.65	100.4	1.64	7.06	114.2 ± 1.38
6-TG	87.51	5.13	13.39	106.6 ± 2.99	106.54	1.62	6.58	98.75 ± 1.73	99.92	1.19	1.34	98.59 ± 1.18
ALP	81.50	10.07	9.45	104.8 ± 6.45	112.8	1.18	9.84	87.28 ± 1.41	100.1	0.34	7.45	88.43 ± 4.61
MSL	107.1	14.97	14.34	110.2 ± 3.02	106.5	3.04	5.34	87.28 ± 1.83	100.3	1.18	7.55	102.7 ± 2.83
PRE	108.1	13.74	15.84	83.04 ± 2.80	102.2	4.19	7.32	88.17 ± 3.56	100.8	0.35	6.12	94.14 ± 2.43

^a^ Each sample was measured 5 times. ^b^ measured on three consecutive days.
